# *Lemna minor*: Unlocking the Value of This Duckweed for the Food and Feed Industry

**DOI:** 10.3390/foods13101435

**Published:** 2024-05-07

**Authors:** Diana Sosa, Felipe M. Alves, Miguel A. Prieto, Mariana C. Pedrosa, Sandrina A. Heleno, Lillian Barros, Manuel Feliciano, Márcio Carocho

**Affiliations:** 1 Centro de Investigação de Montanha (CIMO), Instituto Politécnico de Bragança, Campus de Santa Apolónia, 5300-253 Bragança, Portugal; dianasosa1411@hotmail.com (D.S.); felipemacedo.alves20@gmail.com (F.M.A.); marianapedrosa@ipb.pt (M.C.P.); sheleno@ipb.pt (S.A.H.); lillian@ipb.pt (L.B.); msabenca@ipb.pt (M.F.); 2 Laboratório Associado Para a Sustentabilidade e Tecnologia em Regiões de Montanha (SusTEC), Instituto Politécnico de Bragança, Campus de Santa Apolónia, 5300-253 Bragança, Portugal; 3 Nutrition and Bromatology Group, Department of Analytical and Food Chemistry, Faculty of Food Science and Technology, Universidade de Vigo, Ourense Campus, E32004 Ourense, Spain; mprieto@uvigo.es; 4 Centro de Biotecnologia e Química Fina (CBQF), Universidade Católica Portuguesa, 4169-005 Porto, Portugal

**Keywords:** *Lemna minor*, duckweed, novel food

## Abstract

Duckweed (*Lemna minor* L.) is a small floating aquatic plant that has an important economic impact in several industrial areas. With its high biomass production, reasonable protein content, and resilience to several climates, it has been attracting increasing interest for potential use in animal and human food systems. Historically consumed in southwest Asia, this duckweed is now gaining attention as a potential novel food in Europe. This manuscript explores the contributions of duckweed to various food and feed industries, including aquaculture and livestock, while also pointing out the incipient research carried out for human consumption. Most importantly, it highlights the potential of *Lemna minor* as a vegetable for future human consumption whether eaten whole or through extraction of its nutrients.

## 1. Introduction

The increase in commodities and services production to meet the varied consumption needs of the population is an ever-growing challenge, particularly considering the environmental concerns regarding climate change [1]. The largest consumption of protein in developed countries is through animal-based proteins; however, this production involves direct and indirect environmental impacts, such as changes in soil properties, increase in greenhouse gas emissions, nutrient excretion, and the excessive use of veterinary products that promote the contamination of surface and groundwater. Indirect impacts are associated with the use of fossil energy for production and intercontinental transport of feed, deforestation, and hydric footprint during slaughtering and industrialization [2].

About 30% of climate change originates from the food industry [3], which although being a high contributor is essential to society. In 2019, the report published by the Intergovernmental Panel on Climate Change (IPCC) showed that 70% of the world’s freshwater extractions are for agriculture and livestock, estimating that the water footprint of meat is 10.5 m³/kg [4]. Thus, the main alternatives to animal protein for human consumption are naturally plant-based, but complete substitution is still quite distant due to several constraints, namely economic, societal, and technical [5]. Meat alternatives (plant-based) are more sustainable sources of protein, and they can emulate the flavor, texture, color, and nutritional profile of specific types of meats [6]. Most meat substitutions are derived from soy protein due to this plant’s specific nutritional profile, suitability for various organisms, fat absorption (from the proteins) emulsifying capabilities, and mainly its low-cost production [3]. Still, some problems still linger with the use of soy, namely deforestation (for soy plantation), eutrophication, global warming, reduction in biodiversity, soil erosion, acidification and strain to water resources [7]. Beyond these drawbacks, it is common knowledge that soy and maize will not be the definitive source of plant proteins as mycoproteins, algae proteins, seaweed proteins and bacterial proteins will tend to become the sources of proteins that will feed the world. [8]. Among the alternatives mentioned, wheat is also quite controversial, as it involves great environmental impacts (quality of the water, soil and air). Additionally, wheat exhibits a deficiency in lysine, which is a crucial amino acid for protein synthesis. Consequently, it becomes imperative to advocate for dietary diversification, particularly in instances where wheat consumption is predominant. Since the plants used for protein production are intensively watered, they modify the quality of the hydrological cycles, which is a problem that has not received proper attention [9]. As for the soil, the conversion of native vegetation areas for agricultural exploitation, besides altering the biochemical components, is pointed out as one of the main factors responsible for carbon emissions into the atmosphere, effectively contributing to the concentrations of greenhouse gases, especially CO_2_ [10,11,12,13].

Finding ways to produce plant proteins in a sustainable manner while keeping costs low is a defining challenge for the food industry in the coming years [14]. This can be achieved both through sustainable practices in vegetable production but also by studying new and unused species whose production in quantity and quality has a peaceful relationship with the environment.

An alternative to the apparent drawbacks of protein production from land plants is using aquatic plants, including floating plants as solutions. This paper aims to review the literature and research being carried out on *Lemna minor* L. (one of the most used and studied floating plants). Specifically, we investigate the use of *L. minor* as a source of protein production for human consumption as well as further contributions it has made on sustainability while postulating future applications.

### Lemna minor *L.*

Floating plants are, as the name implies, plants that float in slow-moving water characterized by a fast-growing demeanor especially in favorable conditions. Therefore, they are considered weeds; while some are considered invasive, *L. minor* is not [15,16,17]. 

**Figure 1:**
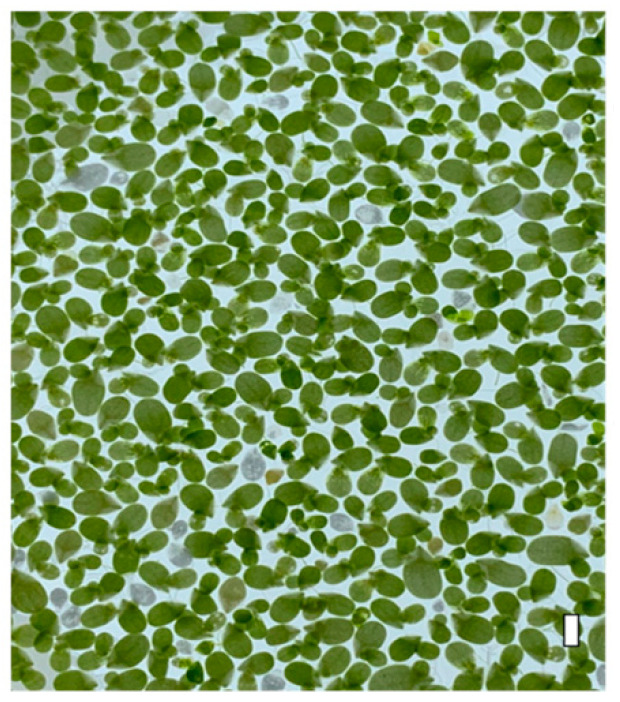
Numerous fronds of *Lemna minor*. Scale bar = 2 mm.

Most floating plants are commonly named duckweeds, and *L. minor* is included in this category. Among the duckweed, Lemna genus, also known as water lentils, stand out. They are tiny fast-growing floating plants with multiple applications [15,17,18,19,20]. This genus represents a small family of floating plants, consisting of 36 species, many of which are feed to ducks, swans, or geese, hence their common name [21,22,23]. The species *Lemna minor* is probably the best known of the genus because of its wide use in research, being one of the smallest among the genus (Figure 1), although being large enough to observe morphological changes with a naked eye [17].

*L. minor* L. was described by Severi & Fornasiero [24] as an aquatic plant of the Lemnaceae family (angiosperm) with an oval shape and an average diameter of 1.7 mm, forming colonies of at least two to four plants and having well-developed root systems. Currently, the taxonomy of this group of plants has undergone some changes, and they are currently classified in the subfamily Lemnaceae within the Araceae family [23,25,26,27].

The estimated production rate of *L. minor* in Europe varies between 7 and 22 tons/ha/year, although most production takes place in India and Central America, mainly for pet food [28]. According to Devlamynck et al. [29], the highest production was achieved in the state of Santa Catarina Brazil (68 tons/ha/year), which was carried out in a pilot scale study in wastewater lagoons from pig-farming activities.

*Lemna minor*, in optimal growth conditions, can duplicate its biomass in a few days, having a wide range of pH and abiotic tolerance [30,31]. The starch content, foliage color, bioactive potential, and protein accumulation, among other parameters, are altered depending on growth conditions, and they can be influenced by environmental parameters (salinity, nutrient deprivation, low or high temperatures, light intensity, and photoperiod) or pollutants (e.g., heavy metals) [32,33,34]. Thus, *L. minor* also has a wide range of acceptable growing conditions, which vary between reports [35,36].

Due to this broad spectrum of growing conditions, resilience and adaptability, *L. minor* has a high potential to complement current and traditional crops as sources of vegetable protein, consistently increasing its outputs [37]. The widespread use as human food is not farfetched, as it has been consumed for decades in southeast Asia, although the European Food Safety Authority (EFSA) in 2015 expressed concerns on using *L. minor* as a new food. The panel of “Nutrition, Novel Foods and Food Allergens” raised concerns on the overexposure of manganese when consuming lentil powder (a mix comprising proteins, fiber and fat of *Lemna* and *Wolffia*) as well as potential allergic reactions due to the high protein content on the plant, although there is no report of toxicity in a 90-day subchronic study in humans consuming 1 mg/kg body weight of *L. minor* [38]. Other duckweeds are already commercialized as human food in the US, EU, Israel, Thailand and Japan, being the species *Wolffia globose* and *Wolffia arrhiza*. In the European Union, the EFSA authorizes the commercialization of some species in accordance with European Parliament regulation 2015/2283, being retailed by private companies. Still, there has been an application to the EFSA for the use of *Lemna minor* and *Lemna gibba* as novel foods, which is still pending [39].

**Figure 2:**
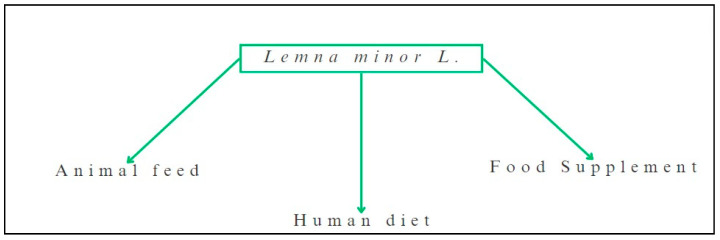
Overview of the different applications of *L. minor* to date.

The US Food and Drug Administration (FDA) has accepted several Generally Regarded as Safe (GRAS) positions for homeopathic medicines based on *L. minor*. And the marketing of a mix of Lemna and Wolffia as a powder ingredient has already been approved and retailed in the US by a private company. Beyond its high applicability, it is a fast-growing plant, although not as fast as another duckweed genus, the Wolffia, which is considered the fastest growing plant in the world by the Salk Institute. Still several scientific outlets postulate *L. minor* as a fast-growing plant with potential to “feed the world”. *L. minor* stands on the verge of becoming a widespread novel food while also being applicable in other sectors of the food industry (Figure 2), yet much of its particularities remain undiscovered.

It also has application in other fields, namely as a phytoremediator and biofuel productor, allowing circularity and the reuse of waste streams, which are essential to improve sustainability [21,22,28,29].

## 2. *Lemna minor* as Feed

### 2.1. Fish Feed

In nature, many fish species feed on these plants, in some cases constituting the base of their diet. A study by Goswami et al. [40] with *Cyprinus carpio* showed five different percentages of *L. minor* being added to their feed (0% (control), 5%, 10%, 5% and 20%), finding that the final weight, growth rate and amino acid content were higher in the fish with diets of 15 and 20% of *L. minor* supplementation. The authors concluded that the inclusion of this duckweed in the diet increased the nutritional value of carp by increasing the contents of proteins, lipids, amino acids, and omega 3. Another study by Irabor et al. [41] using tilapia (*Oreochromis niloticus*) and freshwater shrimp (*Macrobrachium rosenbergi*) showed that 50% replacement of their feed with *L. minor* resulted, after four months, in higher weight gain and faster growth. Another study by Irabor et al. [42] showed that up to 50% of fish feed replacement with *L. minor* is optimal, since above 60%, there is a decline in the growth of catfish (*Clarias gariepinus*). Another study carried out in India by Devi et al. [43] with the objective of determining the protein content of locally available plants, including *L. minor*, tested the in vitro digestibility, simulating the digestion of two fishes *Anabas testudineus* and *Channa punctata*. The highest crude protein was observed in *L. minor*, above 30%, while showing better digestibility for *C. punctata*, around 54%. The relative protein digestibility of *L. minor*, although lower compared to other plant proteins under study, was above 50%, revealing that it can be added to the diet as a substitute for fish protein due to its protein content and high digestibility.

Beyond being used as a staple diet for fish, *L. minor* can also be used as a dried food supplement [21,28,40,42,43] combined with soybeans [44], organic acids [45] and several other types of fishmeal combinations [41,42,46]. As other plants, *L. minor* also synthesizes metabolites from the plant’s secondary metabolism, which for this species remains poorly understood. These molecules, known to have several bioactive effects like antioxidant, antimicrobial and many others, still represent an avenue of research and possible applicability in several industries, namely re-introduction in the food industry, agriculture, pharmacy, supplements, and biomass, among others [20,47,48].

### 2.2. Poultry Feed

Floating plants, and particularly *L. minor*, are also used in poultry diets as substitutes of animal protein. One study focused on the substitution of sesame with *L. minor*, feeding different groups of chickens with increasing amounts of the duckweed, namely 0 (control), 3, 6 and 9%. After six weeks of fattening, the best result was achieved for chickens with 3 and 6% of L. minor in their diet [49]. An experiment in South Vietnam substituted 7% of poultry diet with *L. minor* in 402 recently hatched chickens of a local breed. The diet was combined into treatments with three protein levels: 18, 20 and 22%. The study concluded that animals that were fed with *L. minor* incorporation showed higher weight gain (8.3 g/day) compared to others that were fed the control sample (7.8 g/day). Furthermore, the authors found that the wing and tail feathers appeared first in chickens fed with *L. minor* protein between 20 and 22% [50]. One other study evaluated the growth performance of chickens fed a 0% (control) isostatic diet of *L. minor* plants, 5% with enzyme supplementation and 5% without enzyme, 10% with enzyme and 10% without enzyme. Higher body weight was recorded in the control treatment and the 5% with enzymes, ranging from 37.87 to 41.66 g/day. The highest feed consumption was recorded in the control treatment and the lowest was recorded in the 10%, *L. minor*-based diet with enzyme supplementation [51], implying more satiety from the animals fed with the plant. *Lemna minor* and *Ipomea aquatica* (water spinach) were used as a supplement to the rice-meal based diet of ducks. One group was fed 40% of each compound, while another group was fed a mixture of 35% spinach, 45% *L. minor* and 20% rice feed. The average daily gain and feed conversion were higher for ducks fed *L. minor* as a supplement and lower when fed a spinach-based diet [52]. In the same region, in 2012, another experiment made by Tu (2012) was conducted with 72 ducks, which were fed for 84 days with experimental diets of rice bran and soybean (control), rice bran and high protein *L. minor*, and another group, rice bran with low protein from *L. minor*. The final weight and the daily weight gain were higher with the increase in *L. minor* in the feed while also showing a more attractive carcass skin color for the ducks fed with this diet. The authors also highlighted improvements in the meat as well as economic benefits resulting from using *L. minor* [53]. Overall, the results of *L. minor* as poultry feed seem promising both in terms of sustainability and economics.

### 2.3. Lemna minor in Pig Husbandry

As with poultry, *L. minor* is also used in husbandry, mainly in pigs, as a substitute of other feeds. According to the review article by Sońta et al. [54], the incorporation is well accepted by the animals, allowing for improvements to their carcasses, greater increase in meat and skin, and decrease in fat. However, the feed conservation rate does not have much difference when compared to other substitutions, such as soy, fish meal, and sorghum. Another study by Rojas et al. [55] stated that there were no published data on the nutritional value of Lemna Protein Concentrate (LPC) fed to pigs, and in their study, they conducted three experiments that determined the standardized total tract digestibility (STTD) of phosphorus (P) and standardized ileal digestibility (SID) of AA in LPC and compared these values to those for fishmeal and soybean meal (SBM). They concluded that STTD tended to be higher in LPC than in SBM, which may be justified by the low phytate concentration between them. Amino acids are relatively well digested by younger pigs, and for SID, there was no difference between fishmeal and LPC, while for some indispensable amino acids, it was higher in fishmeal.

When analyzing the total apparent digestibility of dry matter (MSD), crude protein (CPD), neutral detergent fiber (FDN), acid detergent fiber (ADFD), and retained nitrogen (RN) of *Lemna minor*, *Brachiaria mutica* and *Ipomoea aquatica*, added to a basal diet of 8 castrated growing male pigs, Nguyen et al. [56] noted that the males that received a basal diet of 4% of their body mass plus forage showed a higher amount of dry mass from *L. minor* and *I. aquatica*, which were also the most palatable. Furthermore, *L. minor* showed the highest value RN.

### 2.4. Lemna minor in Ruminants

Literature on the use of *L. minor* in ruminants is also scant, although a review paper by Sońta et al [54] studied several types of duckweed, such as *Spirodela*, *Lemna* and *Wolffia*, demonstrating that the dry matter and crude protein are highly degradable in the animal rumen, but the author stressed the need for further research to evaluate the level of supplementation from duckweeds. Another study by Damry et al. [57] determined the quantity and characteristics of wool from Merino ewes (wool yield, rate of wool elongation, fiber diameter) that ingested duckweed and other sources of proteins. When compared, the group fed with *L. minor* showed a higher elongation rate and higher wool volume. Furthermore, the authors considered the protein from *L. minor* as a good “escape protein” for ruminants by analyzing the ammonia concentration in the rumen of the animals. Reid et al [58] studied four different groups of Boer goats gradually fed duckweed substituted for soybean meal. At the end of the experiment, no differences were observed between the groups in nitrogen intake and excretion, serum urea nitrogen level, and phosphorus. The research showed that duckweed is nutritionally able to compare with soybean, and no adverse effects on rumen pH, amount of ammonium ions, and volatile fatty acids were observed with its ingestion. Overall, the applications of *L. minor* in ruminants seems promising, although not much research has been conducted on this husbandry section.

### 2.5. Lemna minor in Human Consumption

When it comes to the use of duckweed in the human diet, even though it has been consumed in some regions of the world for many years, there are still few studies [59]. Still, one of the most important is by Appenroth et al. [17], who analyzed five species of duckweed genera (*Spirodela*, *Landoltia*, *Lemna*, *Wolffiella* and *Wolffia*): namely, their protein, fat and starch content and the distribution of amino acids and fatty acids. Among these plants, the most recommend were Wolffia species, which showed the highest contents of indispensable amino acids for human nutrition.

*Lemna minor*, besides being a rich source of protein, is also known for its secondary metabolic repertoire. According to the literature, its extract has antioxidant and anti-inflammatory activity as well as also being immunomodulatory [31]. Sree et al. [59] corroborated the initial idea of a good qualitative and quantitative profile of the nutritional effects of duckweed in the human diet. They analyzed extracts from the five genera of the family for cytotoxic effects on human cell lines (HUVEC, K-562 e HeLa), as well as anti-proliferative activity, excluding any harmful effect on its consumption, as no anti-proliferative or cytotoxicity was detected.

The work of Beukelaar et al. [60] explores how western consumers perceive *L. minor* and in what context it would be accepted or not. Two questionnaires were conducted, one in-person interview (n = 10) and one online (n = 669), where in both, information was provided on its nutritional and sustainability benefits. Of the two, the online interview was more successful in gaining acceptance for the human diet, but in general, the meal fit consumer expectations, under the assumption that the sensory properties, such as taste, would be satisfactory, and that there would be no major objections to its introduction into consumption. Recent studies have already been comparing the taste of *L. minor* with other vegetables and assessing its health effects. A particular study by Mes et al. [61] compared the taste of *L. minor* and spinach in several dishes over 11 consecutive days with 24 humans. The authors stated that compared to spinach, *L. minor* dishes did not show great differences; the only difference was in the mouth feel of one dish, meaning its acceptability was quite good. Furthermore, in terms of health issues, the authors stated that the consumption of *L. minor* until 170 g per day for 11 days did not show any adverse effects. Table 1 outlines several studies that have been carried out over the years, using *L. minor* and other duckweed species as substitutes in animal feed, as well as preliminary studies as human feed. Regarding *L. minor*, Table 1 shows the different uses of this duckweed in different animal diets, including the species in which it was introduced and the main outcomes.

### 2.6. Lemna minor as a Nutritional Food and Bioactivity

Table 2 highlights the few reports that exist on duckweed, and especially on *L. minor* as human food, including the main results and comparisons with other diets available. As there are very few reports on *L. minor*, articles including other duckweeds (Lemna genera) were included. Other studies have highlighted the anti-nutritional values such as oxalate and phytate, showing moderately high values. Still, several vegetables have high oxalate contents, and there are several methods to reduce this organic acid in foods, which can easily be applied for *L. minor* [62]. Catelani et al. [30] tried to demonstrate the immunomodulatory activity of Lemna extracts by testing them on human immune cells (CD4+, CD8+, B), but they failed to confirm that the extracts were cytotoxic and did not cause cell necrosis or apoptosis. 

Zeinstra et al. [64] examined the postprandial responses of amino acids, glucose, and insulin in adult humans following a single ingestion of Lemna minor compared to peas. However, there was a significant difference in amino acid content between them, and the protein absorption from *Lemna minor* was relatively low. This observation might be explained, in part, by the presence of anti-nutritional factors, such as those mentioned by Ifie (2020) [62]. Table 3 shows the few bioactive studies that were carried out on *L. minor* and other species of the Lemna genera.

### 2.7. Lemna minor Nutritional Profile

The nutritional profile of *L. minor* is shown in Table 4, based on reports of the European Food Safety Authority (EFSA) [68], in g/100 g. EFSA plays a central role in assessing and recognizing novel foods in the European Union (EU), with a science- based approach, meaning that no decision can be made without scientific evidence being available [39]. It has already recognized some duckweeds as novel foods, namely *Wolffia arrhiza* and/or *Wolffia globosa* as a traditional food from a third country under Regulation (EU) 2015/2283 of the European Parliament and of the Council, and amending Commission Implementing Regulation (EU) 2017/2470. But the use of *Lemna minor* is still pending application, when cultivated by water washing and heat treatment, to general population consumption [69]. 

## 3. Final Remarks and Perspectives

Feeding the growing worldwide population is a daunting task, but feeding it in the most sustainable manner is a rather near impossible one. Proteins are vital to the human diet, and their production is now known to be one of the biggest contributing factors to greenhouse gas emissions and consequently global warming, which leads to climate change. While vegetable proteins are somewhat the successor of animal-based proteins, the intensive use of soy or wheat still represents a great unsustainable footprint.

Thus, duckweed, and particularly *Lemna minor*, can be a potential alternative for vegetable-based proteins. Its high biomass production, resistance to several abiotic conditions, small size, and high protein value, but most importantly the fact that it can be grown in recirculating water and/or vertical farming, makes it an ideal candidate to be used in the human diet.

Consumed throughout Asia and accepted by the FDA, *L. minor* needs many more studies on its acceptability as a food or as a source of nutrients, as well as analyzing the different means of production that alter their final nutritional composition. It has proved its worth in animal feed and should slowly be introduced in the human diet. It could also have great contributions to the SDGs, namely goal 2 (Zero Hunger and Sustainable Agriculture) for its use to feed humans [30,31] besides contributing positively to husbandry; goal 12 (Responsible Consumption and Production) since it presents less impact on the soil during production and goal 13 (Action Against Global Climate Change).

The next few years will see pressure put on the current food streams, and the need to find alternatives to the vegetables consumed today will increase. Thus, it will not be surprising if in the next five years, *L. minor* is found in the shelves of supermarkets either as a bulk or its nutrients used in food preparations, pending the extensive research and safety guarantees that are, quite reassuringly, just around the corner. Future research on *L. minor* should base itself on four levels of urgency. The first must focus on improving and optimizing its growth as well as the conditions that favor its development. Published scientific literature on the use of *L. minor* for human consumption is scarce, and each publication varies in terms of the optimal biotic and/or abiotic conditions. Thus, an in-depth, coherent, and reputable research should harmonize the growing conditions. While there are only few publications on this plant, there is knowledge on cultivating it, but it is either kept as industrial secrets or not published, as the industrial production of *L. minor* for human consumption and animal feed is well established. The second level is to increase its protein production though the manipulation of its nutrient source and growth conditions, as was completed with several plants over the course of centuries. Using the technology available today, this task could be accomplished in just a few years. The third level is the reduction in drawbacks associated with the plant, namely palatability issues. While it is known that the plant does not have a very appealing taste when consumed directly, it can be improved in the production phase by reducing certain nutrients or elements that could help the final stage: consumer acceptability. Convincing consumers to eat new foods and new sources of the typical foods they eat is becoming easier, as more consumers are aware of their health benefits. So, showing *L. minor* to more consumers and carrying out consumer acceptance studies is aided by the several benefits this plant can have to the consumer but most importantly to the environment.

## Figures and Tables

**Table 1:** Substitution of animal diets with *L. minor*.

Use	Species Tested	Substitution Quantity	Results	Ref.
Fish Diet	*Cyprinus carpio*	Feeding w/*L. minor*, 0%, 5%, 10%, 15% and 20%	Nutritional value increase in carp flesh by increasing the contents of proteins, lipids, amino acids, and omega 3 fatty acids	[40]
	*Oreochromis niloticus* and *Macrobrachium rosenbergi*	Replace of 50% of their usual ration w/*L. minor*—4 months	Obtained greater weight gain and rapid growth	[41]
	*Clarias gariepinus*	Meal inclusion levels 0%, 20%, 40%, 60% and 80%—56 days	Fish feed replacement with *L. minor* is optimal at 50%, since above 60% there is a decline in growth of catfish species	[42]
	*Anabas testudineus* and *Channa punctata.*	Not available	The highest crude protein was observed for *L. minor*; highest digestibility for *Channa punctata*	[43]
Poultry Diet	Different groups of chickens	Substitution (ad libitum) of sesame oil cake with *L. minor*—42 days old	Partial replacement with *L. minor* in the diet has higher profitability	[49]
	402 baby chicks	Three protein levels, being 18, 20 and 22%	Animals that were fed with *L. minor* incorporation showed higher weight (8.3 g/day) compared to the ones fed the control sample (7.8 g/day)	[50]
	Growing chicks	0% *L. minor*, 5% *L. minor* with enzyme and 5% without, 10% *L. minor* with enzyme and 10% without	Higher body weight was recorded in the control treatment and the 5% *L. minor* with enzymes (37.87 to 41.66 g/day). The highest feed consumption was recorded in the control treatment and lowest in the 10% *L. minor* based diet with enzyme supplementation	[51]
	Ducks	1 group—40% of each compound. 2 group—mixture of 30% spinach, 45% *L. minor* and 20% rice feed	The average daily gain and feed conversion were higher for ducks fed *L. minor* as a supplement, and lower when fed a spinach-based diet	[52]
	Ducks	Experimental diets of rice bran and soybean, rice bran and high protein *L. minor*, and rice bran with low protein *L. minor*—84 days	The final weight and the daily weight gain were higher with the increase in *L. minor* in the feed, while a more attractive skin color was also apparent for the ducks fed with this diet, improvements in the meat of the ducks and economic benefits resulting from using *L. minor*	[53]
Pig Husbandry	Pigs	Not available	Incorporation well accepted by the animals, allowing for improvements to their carcasses, greater increase in meat and skin, and decrease in fat	[54]
	3 groups of pigs	Experiment (Exp.) 1—fish meal, soybean, and corn. Exp. 2—*L. minor*, fish meal and soybean. Exp. 3—*L. minor* and fish meal	The standardized total tract digestibility of phosphorus tended to be higher in *L. minor* diet than in soybean. Amino acids are relatively well digested by younger pigs; there was no difference between fishmeal and *L. minor*	[55]
	Exp. 1—4 crossbred (Yorkshire x Baxuyen) castrated male pigs (53 kg). Exp. 2—the same but with 70 kg	Exp. 1—16% crude protein + *L. minor*, Para grass and water spinach. Exp 2—14% crude protein + *L. minor*, Para grass and water spinach	*L. minor* showed the highest value of N retained. *L. minor* and *I. aquatica* were also the most palatable. Replacing the basal diet at a high rate, the water content in the plants is an important limitation, reducing feed intake	[56]
Ruminants	Merino (ewes)	Exp. 1—Edible straw—50 g for E1, 100 g for E2 and 1 kg for E3, flour for control Exp. 2—urea for control, cottonseed meal for E1 and duckweed for E2	For the first experiment case, the parameters did not diverge from each other. For the second, the control had a slower elongation rate and lower wool yield than the other two, determining duckweed as a valuable protein source	[57]
	Boer goats	Gradual substitution of soybean by duckweed	No differences were observed between the groups (nitrogen intake and excretion, serum urea nitrogen level, and phosphorus). The ingestion of *L. minor* had no adverse effects on rumen pH, amount of ammonium ions, and volatile fatty acids	[58]

**Table 2:** Uses of *L. minor* in human diets.

Use	Species Tested	Substitution Quantity	Results	Ref.
Human consumption	Not available	Protein value, fat and starch content were analyzed, along with the distribution of amino acids and fatty acids	The most recommended were of the Wolffia species, because in the six species investigated, *W. microscopica* and *W. hyalina* showed the highest contents of amino acids indispensable for human nutrition	[15]
	Not available	Compared the taste of *L. minor* and spinach in several dishes over 11 consecutive days with 24 humans	The consumption of *L. minor* until 170 g per day for 11 days did not show any adverse effects. The authors finish by saying that due to wide consumption in Asian countries, and acceptance by the FDA, the European Union could eventually accept it as a novel food	[61]
	Not available	Postprandial and overnight glycemic response using an iso-carbohydrate/protein/caloric dairy shake	When compared with the yogurt group, it showed a beneficial glycemic response. The individual pattern is more pronounced than the intervention itself. Duckweed can serve as an emerging substitute source of plant protein with promising potential postprandial glycemic effects	[62]
	Not available	Explore the availability of essential amino acids (EEA) through duckweed intake in apparently healthy men by comparing iso-protein intake against established animal (soft cheese) and vegetable (peas) protein meals	After duckweed consumption, the blood concentration of EEAs was similar in animal and plant protein sources and may be a plant-compatible protein source for meat exclusion	[63]

**Table 3:** The bioactivity and nutritional value of *L. minor*.

Application	Analysis	Results	Ref.
Bioactivity	Not available	According to the literature, its extract has antioxidant and anti-inflammatory links as well as immunomodulatory activity	[30]
	Cytotoxic effects on human cell lines (HUVEC, K-562 e HeLa), as well as anti-proliferative activity	No anti-proliferative or cytotoxicity detected	[59]
Nutritional	Analyze the anti-nutritional values such as oxalate and phytate	The average oxalate and phytate content determine that bleaching with sun drying can be employed to concentrate the nutrients in the *L. minor*	[63]
	Explores how Western consumers perceive duckweed and in what context it would be accepted or not	The meal fits the consumer’s expectations under the assumption that the sensory properties, such as taste, would be satisfactory, and that there would be no major objections to its introduction into consumption	[60]
	Antioxidant analysis by three methods—DPPH, ABTS and FRAP	Antioxidant activity was lower than in other studies using fruit, but TPC and TFC tended to be like other vegetables. Phenolics and flavonoids have been shown to contribute greatly to antioxidant activity	[65]
	Not available	Duckweed species harbor numerous vital nutrients, including significant levels of protein and dietary fiber, ample essential amino acids, health-enhancing micronutrients, and antioxidants. The genus of Wolffia contains a high content of lutein, β-carotene, α-tocopherol, and zeaxanthin	[66]
	The antioxidant activity was made in vitro and were expressed as µg trolox equivalents (TE)/mL in the DPPH•, ABTS•+, CUPRAC, and FRAP assay	*L. minor* was a rich source of carotenoids and total flavonoids (mainly flavones and flavonols), followed by phenolic acids, low-molecular-weight phenolics and glucosinolates.	[67]

**Table 4:** Nutritional profile of *L. minor* in g/100 g.

Parameter	Specification
Moisture	91–95 g/100 g
Proteins (N × 6.25)	1–4 g/100 g
Carbohydrates	1–3 g/100 g
Dietary fiber	0.5–3 g/100 g
Ash	1–2 g/100 g
Fat	0.2–0.6 g/100 g
Oxalates (as calcium oxalate)	<1.6 g/kg
Beta-Carotene	<3160 µg/100 g
Folate	<38 µg/100 g
Phylloquinone	<46 µg/100 g
Copper	<2.5 mg/kg
Iron	<53 mg/kg
Manganese	<18 mg/kg
Molybdenum	<0.5 mg/kg
Zinc	<20 mg/kg
Chromium	<1 mg/kg
Boron	<15 mg/kg

Source: Turck, D et al. [69] —EFSA Journal.

## Data Availability

No new data were created or analyzed in this study. Data sharing is not applicable to this article.
